# Purification and Characterization of Bioactive Metabolite from *Streptomyces monomycini* RVE129 Derived from the Rift Valley Soil of Hawassa, Ethiopia

**DOI:** 10.1155/2022/7141313

**Published:** 2022-12-20

**Authors:** Firew Elias, Sudhamani Muddada, Diriba Muleta, Belachew Tefera

**Affiliations:** ^1^Koneru Lakshmaiah Education Foundation, Department of Biotechnology, Vaddeswaram, Guntur, India; ^2^Ethiopian Agricultural Authority, Animal Products, Veterinary Drug, and Feed Quality Assessment Centre, Ethiopia; ^3^Environmental Biotechnology Unit, Institute of Biotechnology, Addis Ababa University, Ethiopia

## Abstract

*Streptomyces* species have produced a variety of bioactive secondary metabolites with intriguing antimicrobial and anticancer properties. In this study, the bioactive compound obtained from the potent strain RVE129 was purified and characterized. Its bioactivity against various pathogens and its cytotoxicity toward the human cervical cancer (HeLa) cell were also examined. The strain was previously isolated from unexplored areas of the rift valley soil of Hawassa (Ethiopia) and identified by phenotypic characteristics and complete sequencing of the 16S rRNA gene and found to be closely related to *Streptomyces monomycini* strain NRRL B-24309 (99.65%); accession no. (ON786620). The active fraction undergoes bioassay-guided purification using the TLC method after being extracted by ethyl acetate. Then, it was subjected to physicochemical and structural characteristics using UV–Vis, FTIR, and NMR spectroscopic methods. A minimum inhibitory concentration of the purified antibiotic was achieved by the broth microdilution method. The cytotoxicity of HeLa cells was determined using the 3-(4,5-dimethylthiazole-2-yl)-2,5-diphenyltetrazolium bromide (MTT) assay. The acquired data from spectroscopic studies was compared with that of the reported natural compounds in data bases and found to be the known antibiotic, setamycin. The antibiotic (RVE-02) showed a broad spectrum of bioactivity against both Gram-positive and Gram-negative bacteria, with MIC values that ranged from 1.97 to 125 *μ*g/ml. The bioactivity results also demonstrated antiproliferation and morphological change in HeLa cells with an IC_50_ value of 24.30*μ*g/ml of antibiotic. The antibiotic, obtained from *S. monomycini* RVE129, could be a potential candidate to combat pathogens including drug-resistant *S. aureus*. Further, the effect on HeLa cells suggests that it could be a prominent cancer chemotherapeutic agent.

## 1. Introduction

The rapid emergence of resistant pathogens to multiantibiotics along with the limited availability of effective anticancer drugs has led to the urgent need for focused research on effective antimicrobial and anticancer agents with minimal undesired side effects [1]. Microorganisms have been used as a promising source for developing a variety of bioactive metabolites that can be used to fight the spread of infection, deadly diseases such as cancer, and the toxicity associated with currently used cancer drugs [1–3]. Diverse potentially significant species have been researched as a natural source for the production of novel bioactive compounds for the treatment of allergies, autoimmune diseases, infections, cancer, inflammatory diseases, and others [4, 5]. Actinomycetes have been extensively explored and utilized as a group of microorganisms for the development of antibiotics and other important natural bioactive molecules [5]. Among actinomycetes, *Streptomyces* species are the most potent reservoir of many novel compounds with a broad array of bioactivities [6, 7].

Currently, in the route of selection for novel bioactive molecules, many researchers are concentrating on isolating actinomycetes from uncharted and uncommon environments to combat the spread of infectious diseases by pathogens [6] and cancer [7, 8]. However, the number of new microbial natural products has been declining due to the increasing rediscovery of old known molecules. In exploring the poorly studied and unique habitat with varied climatic zones of the Rift Valley of Hawassa, Ethiopia, a promising actinomycete strain, RVE129, belonging to the genus *Streptomyces* sp., was selected and studied.

A few studies have been reported in Ethiopia on the antimicrobial activity of actinomycetes, but no published evidence exists on the antitumor activity. In light of these observations, the current research aims at the purification and characterization of antibacterial compounds as well as evaluating their bioactivity against drug-resistant pathogens. The cytotoxic activity of the bioactive compound RVE-02 was also investigated *in vitro* on human cervical carcinoma (HeLa) cells.

## 2. Materials and Methods

### 2.1. Microorganism and Maintenance

The potential antimicrobial metabolite-producing actinomycete isolate, *Streptomyces* RVE129, was previously recovered from the rhizosphere soils of the Rift Valley areas of Hawassa, Ethiopia [9]. After isolation, the strain was kept in a pure culture over starch casein slants and stored at 4°C. Different bacterial test strains, including *Staphylococcus aureus* ATCC 259233, *Staphylococcus epidermidis* ATCC 12228, *Klebsiella pneumonia* ATCC 700603, *Pseudomonas aeruginosa* ATCC 27853, *Salmonella typhi* ATCC 13311, *Salmonella typhi* ATCC 14028, and *Escherichia coli* ATCC 25922, were supplied by the Ethiopian Health and Nutrition Research Institute (EHNRI). All bacterial strains were maintained in the refrigerator on Mueller-Hinton's agar slants.

### 2.2. Morphological and Cultural Characterization

The macromorphology of the strain was visually examined to determine the growth pattern, substrate, and aerial mycelia as well as the colour of diffusible pigment by culturing on starch casein agar [10] and International *Streptomyces* Project (ISP) media plates as suggested by the ISP and compared with Bergey's manual [11]. The pure culture of the selected potent isolate was spread on the agar plates of the international *Streptomyces* project (ISP) medium plates as recommended by the ISP, compared to Bergey's manual, and incubated for 10 days at 30°C [12]. Micromorphological features such as spore morphology and hyphae were identified by the coverslip method and the Gram staining method using a well-grown sporulated culture plate in yeast extract malt extract agar medium plate at a 45° inclination using a trinocular light microscope at 1000x magnification, and the spore shape and surface were determined by using a scanning electron microscope (Cambridge Stereoscan, model 240) according to the method of Singh et al. [13].

### 2.3. Physiological and Biochemical Characterization

Physiological tests such as different temperatures (15, 20, 25, 30, 37, and 45°C), pH range (4, 5, 6, 7, 8, 9, 10, 11, and 12), and NaCl concentrations (0, 2.5, 5, 7.5, and 10%) for the cultivation of RVE129 isolate were evaluated on ISP2 agar plates after two weeks of incubation. Sugar utilization tests were determined by growth on the ISP-9 medium, added with 1.0% of each of the carbon sources at 30°C as described by Shirling and Gottlieb [11]. The isolate was screened for reduction of nitrate, gelatin degradation, starch hydrolysis, casein, catalase, urease, and H_2_S production and were determined according to Locci [12], and melanin production was evaluated on tyrosine agar (ISP 7) medium [14].

### 2.4. Molecular-Based Analysis

The amplification and sequencing were carried out by the Microbial Type Culture Collection and Gene Bank in Chandigarh, India. Taxonomic identification of the strain was further verified by performing PCR reactions using universal primers for amplification and subjecting them to the 16S rRNA Genomic DNA gene sequence analysis [15]. The standard basic local alignment search tool (BLAST) program was used to match partial DNA sequences of 16S rRNA from the public database (NCBI) [16, 17]. The 16S RNA gene sequence was compared to other relevant sequences using the Clustal W, and the BLAST search technique was employed to examine for similarities [17]. Individual accession numbers were assigned after the sequences were deposited in the NCBI gene database. Phylogenetic tree construction was conducted using a neighbor-joining approach by MEGA software version 6.0. The isolate and related type strains' representative nucleotides from the total and partial 16S rRNA sequences in the GenBank database were used to generate the tree from the high similarity sequences.

### 2.5. Production and Extraction of Bioactive Metabolites

Production of active metabolites from the potential *Streptomyces* RVE129 sp. isolate followed the shake flask fermentation approach as described by Oskay [18] and Singh et al. [13] with a few modifications. In brief, the seed culture was obtained by inoculating spore suspensions of RVE129 in ISP2 broth medium and incubating for 3 days at 30°C on a shaking incubator (150 rpm). Following incubation, 5% of the culture was inoculated into 200 ml of cultivation medium containing 10 g starch, 2 g NaCl, 2 g (NH_4_)_2_SO_4_, 1 g MgSO_4_.7H_2_O, 1 g K_2_HPO_4_, and 20 g of agar in 1.0 litre of distilled water. The shake flask was placed in the shaking incubator (150 rpm) for 10 days at 30°C. The fermentation broth was centrifuged at 10,000 rpm for 15 minutes and filtered through Whatman No. 1 to remove the mycelial biomass. The culture filtrates and the ethyl acetate solvent were mixed at a 1 : 1 ratio (w/v) and agitated continuously for 60 min. The organic phase was evaporated and concentrated under a rotary evaporator at 50°C and stored at 4°C. Finally, the dried bioactive metabolite was accumulated and preserved at 4°C for further testing.

### 2.6. Purification of Antimicrobial Agent

After organic solvent extraction, the purification of active metabolites was done by thin-layer chromatography (TLC) plates in a mobile solvent technique following the method of Fried and Sherma [19]. Twenty *μ*l of extract (1 mg/mL) was loaded at the bottom of the TLC plate with a capillary tube and placed inside the solvent chamber with a mobile phase of ethyl acetate: hexane (1 : 1). The plates were removed aseptically and allowed to dry at room temperature once the solvent had reached the top of the chromatography board. The spotted TLC plate was kept in a closed iodine chamber to expose iodine vapour and visualized under the ultraviolet light wavelength of 254 nm. The retention factors (RF) values of the different spots were observed and calculated.

### 2.7. Detection of Bioautography

After TLC was performed, the bioautography method was followed as reported by Selvameenal et al. [20] for the identification of the antimicrobial activity of spots separated in TLC. Briefly, TLC strips were placed aseptically on the surface of a sterile Mueller-Hinton's agar (MHA) plate inoculated with 200 *μ*l of the fresh culture cell count (3 × 10^6^) of *S. aureus* ATCC 259233 and *S. typhi* ATCC 14028. Plates were kept in the refrigerator for 1 hour before being incubated at 37°C for 24 hours. Growth inhibition in the region of the spot suggests that it has an active antimicrobial compound.

### 2.8. Identification of Bioactive Compound

#### 2.8.1. Temperature and pH Stability Study

The solubility of purified antibiotics was evaluated using various solvents such as chloroform, methanol, acetone, ethanol, petroleum ether, and water. The melting point of purified compound was determined by the Fisher-Johns melting point apparatus. The characteristics of purified antibiotics derived from *S. monomycini* RVE129 were examined by the standard procedures of Harindran et al.'s [21] slight modification. Briefly, the purified antibiotic sample was placed in test tubes and exposed to various temperatures (0°C, 20°C, 30°C, 40°C, and 50°C) to determine the temperature stability with regard to its antibacterial activity. Then, the solutions were cooled to room temperature and subjected to antibacterial activity tests against *Staphylococcus aureus* by well diffusion assay [22]. The purified antibiotic sample was placed in separate test tubes, and the pH was adjusted to different ranges (4–12) using 0.1 N NaOH or 0.1 N HCl to evaluate pH stability. The tubes were neutralized and left at room temperature for 60 minutes before being evaluated for bioactivity against *S. aureus* using an agar well diffusion assay.

#### 2.8.2. Spectroscopic Studies

The active fraction extracted with methanol from TLC was evaluated with several spectrum studies for the identification of the active compound [23]. The UV-vis spectra of the purified fraction was qualitatively observed on a UV-vis spectrophotometer (Shimadzu, Kyoto, Japan) and scanned over the desired wavelength range of 200 and 400 nm using methanol as a reference solvent. The Fourier transformation infrared (FTIR) spectroscopy analysis of the purified antibiotic fraction was done with KBr pellets. The FTIR spectra were observed using (Shimadzu, Kyoto, Japan) spectroscopy at between 400 and 4000 cm^−1^ wavelength. The results of FTIR spectrum patterns are analyzed and matched to determined compounds in the FTIR library [24]. Nuclear magnetic resonance (NMR) spectroscopy (Bruker DRX500, Germany) was used to record the ^1^H and ^13^C NMR spectra of the purified antibiotic in DMSO at 500 MHz for ^1^H and 150 MHz for ^13^C. To calibrate chemical shifts in ^1^H and ^13^C NMR spectra, an internal standard of 0.1% (v/v) tetramethylsilane (TMS) was used.

### 2.9. Biological Assays

#### 2.9.1. Antimicrobial Assay

The bioactivity of isolated bioactive compounds from *Streptomyces* sp. RVE129 against several test pathogens was investigated using the standard reference methods of the Clinical and Laboratory Standards Institute [25]. A stock solution (50 mg/ml) of the active purified fraction was scrapped from TLC, prepared in 50% dimethyl sulfoxide (DMSO), and mixed with Mueller-Hinton's broth (MHB) to working concentrations, whereas the antibiotics were dissolved in sterile distilled water. A bacteria culture was standardized at 0.5 McFarland and diluted 1 : 100 in saline to have a final cell count of 1 × 10^6^ CFU/mL. The bioactive compound and standard antibiotics (streptomycin) at concentrations (1.97, 3.95, 7.56, 15.12, 31.25, 62.5, 125, and 250 *μ*g/ml) were prepared with sterile water using two-fold serial dilutions. Each well of a microtiter plate was filled with one hundred microliters of test concentration of the compound along with the microbial suspension and 100 *μ*L of sterile MHB. A control was established using MHB media containing cultures of test microorganisms with 50% DMSO as a control. The MICs were determined by incubating microtitre plates for 24 hours at 37°C. The lowest concentration of the test fraction that inhibited observable bacterial growth was determined as the MIC value.

#### 2.9.2. In Vitro Cytotoxicity Assay

The in vitro cytotoxicity of purified antibiotics RVE-02 was assessed using a 3-(4,5-dimethylthiazol-2-yl)-2,5-diphenyltetrazolium bromide (MTT) dye cell viability assay [26]. HeLa cells were cultured in Dulbecco's modified eagle's medium (DMEM), which was supplemented with 0.1 mM sodium pyruvate, 1 mM with 10% fetal bovine serum, and 0.5 mM L-glutamine. The cells were cultured in 96-well microplates at a density of 0.2 × 10^6^ cells per well with various concentrations of purified fraction (3.78, 7.56, 16.12, 31.25, 62.5, 125, and 250 *μ*g/ml) and incubated for 24 hours in 5% CO_2_ at 37°C. Then, the medium was treated with 20 *μ*L of MTT solution (5 mg/mL in PBS) in each well and incubated for 4 h at 37°C with 5% CO_2_. Finally, to dissolve the formazan crystal product, 200 *μ*l of DMSO was added to the solution and well mixed. HeLa cells treated with DMSO under the same conditions were used as a positive control. Optical density (OD) at 570 nm was used to determine absorbance after incubation [27, 28]. The following formula was used to calculate cell viability:

(1) $$\begin{array}{c} \text{Viability of cell}\left(\%\right)=\frac{100-\text{Abs}\left(\text{sample}\right)}{\text{Abs}\left(\text{control}\right)}\times 100. \end{array}$$

The half inhibitory concentration (IC_50_) value was determined as a 50% reduction in absorbance, compared to the control experiment. Assessment of the cell morphology was performed by using a phase-contrast inverted microscope (Olympus, Japan), and photographs were captured using a digital camera.

## 3. Results

### 3.1. Cultural, Physiological, and Biochemical Characteristics

**Figure 1:**
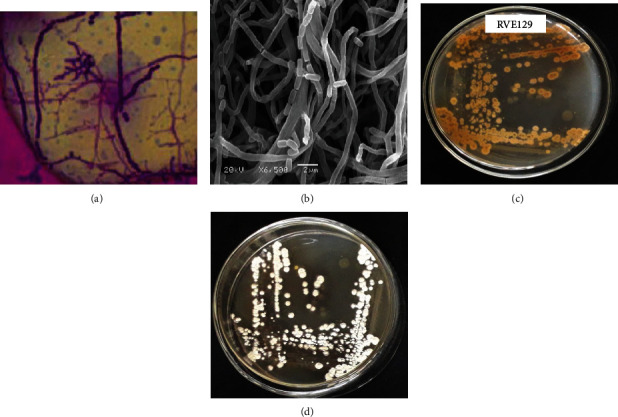
Morphological characterization of *Streptomyces* spp. RVE129 on ISP2 medium. (a) Aerial hyphae bearing spore chains under light microscope (1000x), (b) scanning electron micrograph of strains, (c) substrate mycelium, and (d) aerial mycelium.

The microscopic observation of the isolate, designated as *Streptomyces* RVE129, showed a Gram positive, long aerial mycelium, filamentous, and rectiflexible (RF) in the arrangement. Scanning electron microscopy analysis also showed long smooth straight spores arising from the aerial mycelia and vegetative hyphae (Figure 1). Hence, the isolate was placed in the group of *Streptomyces* sp.

The growth pattern of *Streptomyces* sp. RVE129 was evaluated on different agar media as illustrated in Table 1. The growth pattern of isolate RVE129 exhibited some variations among the tested media. The results showed good growth on starch casein agar, peptone yeast extract iron agar medium (ISP-6), starch-inorganic salts agar (ISP-4), and yeast extract malt extract dextrose agar (ISP-2) in comparison to the other studied growth media (Table 1). The RVE129 strain displayed brown substrate mycelium and white aerial mycelium without diffusible pigment on ISP 2 plate medium (Figure 1 and Table 1).

The biochemical and sugar fermentation test results of strain RVE129 are illustrated in Table 2. The isolate was grown in a pH range of 4–10, a temperature range of 20–40°C, and a NaCl concentration of up to 5% (w/v). The isolate showed optimal growth at 30°C and at pH 7 as well as in the presence of a 2.5% NaCl (w/v) concentration. The isolate could utilize different sugars as carbon sources, although inositol, arabinose, and rhamnose were not utilized (Table 2). The strain RVE129 exhibited positive results for the production of catalase, urease, H_2_S, citrate, hydrolysis of starch, casein, gelatin, and esculin degradation, whereas melanin formation, nitrate reduction, production of indole, Voges-Proskauer, and oxidase were shown negative results (Table 2).

### 3.2. Molecular Characterization

**Figure 2:**
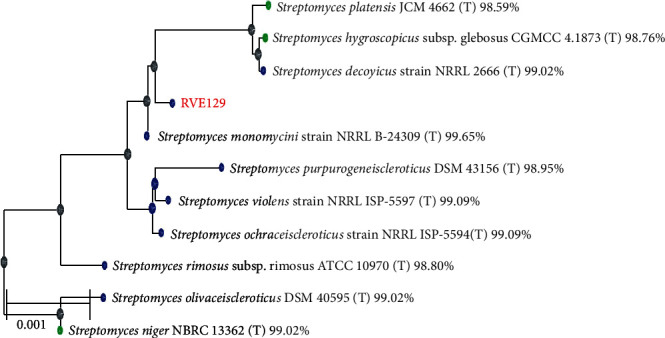
Phylogenetic tree constructed based on 16S rRNA gene sequences of RVE129. The percentage of similarity between the strain and related members of the genus *Streptomyces* 16S rRNA gene clade is shown next to the strain names.

According to the sequencing results, 1435 base pairs were present in the isolate and deposited in NCBI GenBank under the accession number ON786620. https://www.ncbi.nlm.nih.gov/nuccore/ON786620.1. A phylogenetic tree of the RVE129 strain and allied reference strains is shown in Figure 2. Examination of the 16S rDNA gene of the *Streptomyces* sp. RVE129 sequence showed maximum similarity (99.65%) with the 16S rRNA genes of *Streptomyces monomycini* NRRL B-24309^T^ as indicated in Figure 2

### 3.3. TLC Profile of the Antibacterial Extract

**Figure 3:**
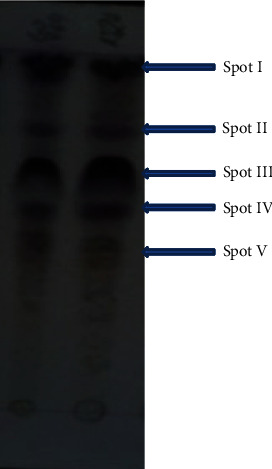
TLC chromatograph of the extract from *Streptomyces* sp. RVE129.

Thin-layer chromatography (TLC) was used to purify the antibacterial component. The separate spots of the bioactive metabolite in the TLC chromatogram are presented in Figure 3. The TLC results show that the extract produced five spots, denoted I, II, III, and IV, with RF values of 0.96, 0.73, 0.63, 0.43, and 0.34, respectively (Figure 3).

The TLC-agar overlay bioautography assay revealed spot II (RF = 0.73) as the active spot against *S. aureus and S. typhi*. The purified antibiotic was denoted as RVE-02.

### 3.4. Characterization of the Active Compound

#### 3.4.1. Physicochemical Characterization

The purified antibiotic RVE-02 was yellowish-brown in colour and had a melting point of 135°C. It was soluble in methanol, ethanol, acetone, chloroform, petroleum ether, ethyl acetate, and DMSO but insoluble in water. The antibacterial activity of purified antibiotic RVE-02 was tested at various temperatures and pH levels, and it was revealed to be stable in the pH range (4–10) and over a wide range of temperatures (20–45°C). The purified antibiotic compound treated at temperatures of 30°C retained maximum antimicrobial activity against *S. aureus* ATCC-259233. The antibiotic RVE-02 was most stable between pH 7.0 and 9.0, with activity decreasing slightly at acidic or basic pH.

#### 3.4.2. Spectral Characteristics of Antimicrobial Compound

**Figure 4:**
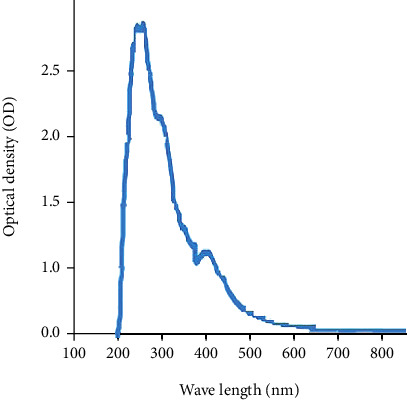
UV spectra of the purified antibiotic RVE-02.

The active fraction (RF = 0.73) obtained from TLC was examined by spectral analysis. The ultraviolet (UV) of antimicrobial compound (dissolved in methanol) showed three characteristic absorption peaks of maximum absorption at 243 nm, 279 nm, and 420 nm and with a shoulder at 327 nm (Figure 4).

**Figure 5:**
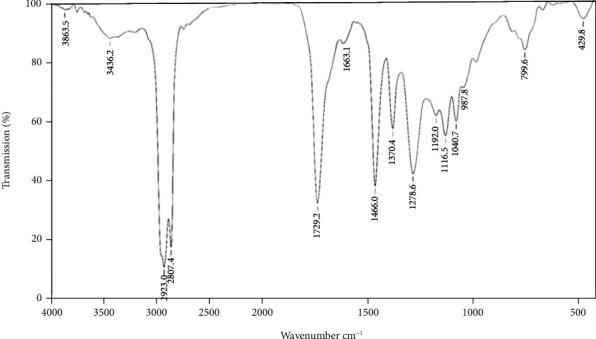
FTIR spectra of purified antibiotic RVE-02.

Infrared spectra (IR) showed peaks at 3863.5, indicating the presence of hydroxyl functional group (-OH). FTIR spectra peak absorbance located in the range of 3240 to 3511.01 cm^−1^, with a maximum peak at 3436.2 cm^−1^ is indicative of amine function and a broad hydroxyl functional group (Figure 5). Analysis of the FTIR spectrum also showed typical absorption peaks at 1663.12 cm^−1^ corresponding to conjugated C=O, indicating the presence of an ester group and the absorption peaks 1729.2 cm^−1^ corresponding to aldehyde. The peak at 1116.58 cm^−1^ indicates the presence of the alcohol functional group. The presence of alkane groups is indicated by a sharp absorption peak at 2923 cm^−1^. The spectrum peak at 987.84 cm^−1^ indicates the presence of methylene compounds with C-C vibrations, and the peak appearing at 797.06–801 cm^−1^ was assigned to *p* disubstituted benzene.

**Figure 6:**
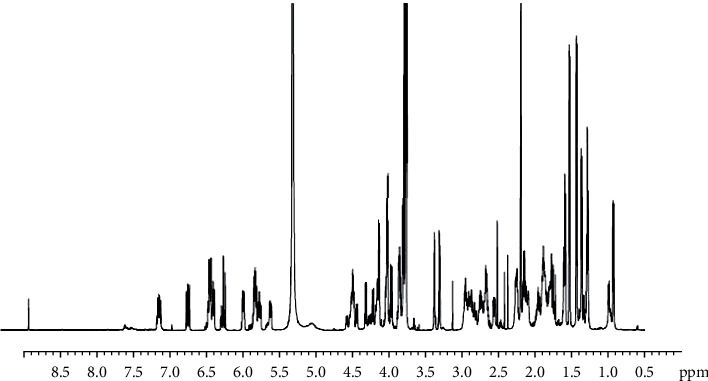
^1^H NMR (500 MHz, DMSO/CDCl_3_) spectrum data of antibiotic RVE-02.

From the ^1^H NMR spectrum data (Figure 6), the signal at *δ* 8.84 (N-H) confirmed the existence of the amide functionality, and the chemical shifts observed between 𝛿 H *δ* 6.5–*δ* 7.27 ppm indicate the presence of aromatic protons. The signals in the range of 𝛿 H, 4.18–4.35 indicates the presence of the terminal long chain alkane in the compound. The presence of oxygenated methine proton is supported by the chemical shift signals in the region 𝛿 H 3.645–3.79 ppm region for the methoxy group. The chemical shift value in the range of 𝛿 H 0.45–2.69 ppm region corresponds to the occurrence of aliphatic hydrogens i.e., methyl and methylene groups in the side chains (Figure 6).

**Figure 7:**
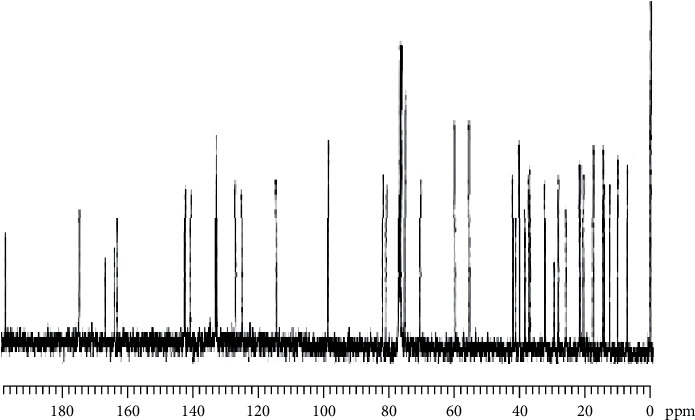
^13^C NMR (500 MHz, DMSO/CDCl_3_) spectrum data of antibiotic RVE-02.

In addition, the ^13^C NMR spectrum (Figure 7 and Table 3) of antibiotic RVE-02 indicated that the signal in the range of *δ*C 15 to 19 ppm corresponds to methyl (-CH_3_) groups and peaks in the range *δ*C 21 to 38 ppm represents the presence of different methylene (-CH_2_-) groups and the peaks in the range *δ*C 39 to 58 ppm a characteristics of methine (CH) group as shown in (Figure 7). The peaks n the range of *δ*C 60-96 ppm represents methoxy groups, and quaternary carbons were detected at *δ*C 100-168 ppm and one *α*, *β*-unsaturated lactone carbonyl at *δ*C 144.3 ppm. Finally, the peak at *δ*C 167 to 198 ppm belongs to carbonyl carbon group of either an amide or an ester (Figure 7).

### 3.5. Biological Assays

#### 3.5.1. Antimicrobial Activity of Purified Antibiotic RVE-02

The minimum inhibitory concentration (MIC) of the bioactive compound RVE-02 is presented in Table 4. The antibiotic RVE-02 demonstrated broad antibacterial efficacy against Gram-positive and Gram-negative bacteria, with MIC values ranging from 1.97 to 125 *μ*g/ml. When compared to streptomycin (1.97 *μ*g/ml), the lowest MIC was recorded against *S. aureus* (1.9 *μ*g/ml) and the highest against *K. pneumonia* and *P. aeruginosa* (125 *μ*g/ml). Although in most of the cases, control antibiotics had a lower MIC value than the RVE-02 antibiotic; it showed good activity (1.97 *μ*g/ml) against *S. aureus* when compared to standard antibiotics (1.97 *μ*g/ml).

#### 3.5.2. In Vitro Cytotoxicity Analysis

**Figure 8:**
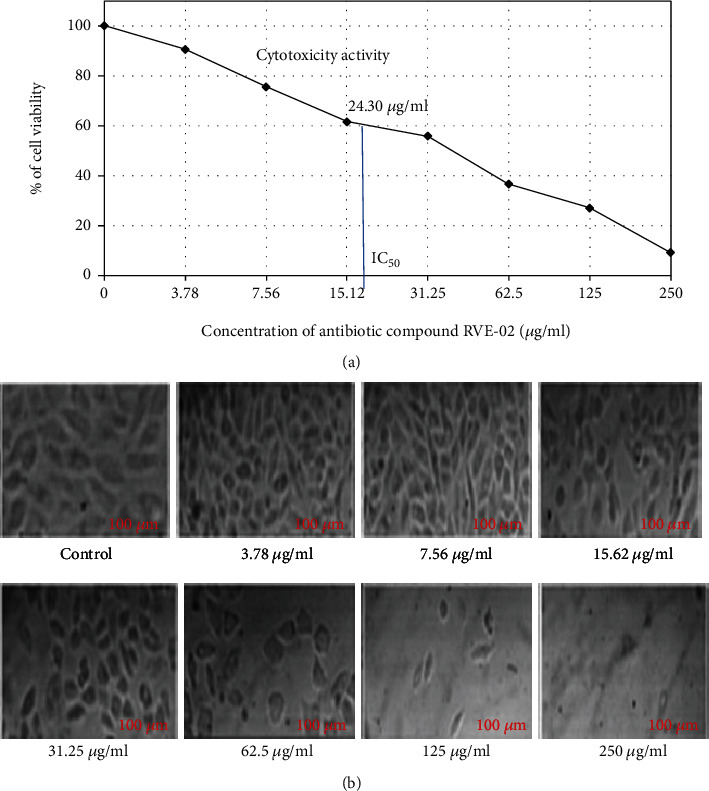
(a) Cytoxicity different concentrations of active metabolite on the percentage of HeLa cell viability. The data are represented as the mean ± SD of three separate experiments, for each experiment. (b) Inverted microscopic (40x) images of HeLa cell lines treated with different concentrations of antibiotic RVE-02.

The results of the cytotoxicity assay are presented in Figure 6. The in vitro cytotoxic experiment showed that the RVE-02 antibiotic obtained from *S. monomycini* RVE129 inhibited cell growth and viability in the human cervical cancer (HeLa) cell line (Figure 8(a)).

The cytotoxicity results showed that the purified antibiotic RVE-02 had a considerable antiproliferative influence on HeLa cells, with an IC_50_ value of 18.30 *μ*g/ml of concentration (Figure 8(a)). The lowest tested concentration (3.78 *μ*g/ml) was able to inhibit cell line proliferation by less than 10%. In contrast, more than 90% of cell line proliferation inhibition was recorded after 24 hours treatment at 250 *μ*g/ml (Figure 8(b)). These results showed that the antibiotic RVE-02 induced cell cytotoxicity against HeLa cell lines in a direct dose-response relationship where the viability of cells was reduced, and a change in the shape of the cell was observed with an increased antibiotic concentration in the treated cells (Figure 8(b)).

## 4. Discussion

The development and spread of multidrug-resistant bacteria that are resistant to currently available antibiotics have become a global challenge [1, 2]. This will threaten our potential to treat common infectious diseases and cancer [3]. As a result, screening for highly efficient, innovative, and novel broad-spectrum antimicrobial metabolites is still urgently needed in the development of antimicrobial metabolites to combat these pathogens. The focus of this research is to purify and characterize a bioactive metabolite produced by *Streptomyces* sp. RVE129, which was previously isolated from the rift valley areas of Hawassa, Ethiopia. The bioactive metabolite was then examined for its bioactivity against various pathogens, including drug-resistant opportunistic pathogens and for its cytotoxicity against the human cervical cancer (HeLa) cell.

Furthermore, in accordance with Barka et al. [5], the genus *Streptomyces* can be distinguished from all other bacterial groups by a broad range of stable exhibited phenotypic features such as morphology, physiology, and biochemical traits. The RVE129 strain was aerobic, Gram positive, exhibited good growth on different growth medium, aerial, and substrate mycelia of different colours which are typical characteristics of actinomycete. The features of strain RVE129 were compared with those of actinomycete species noted in Bergey's manual of determinative bacteriology [12], and obtained characteristics strongly suggested that strain RVE129 belonged to the genus *Streptomyces.* The strain assimilated the majority of the tested sugars, demonstrating a diverse pattern of carbon utilization. These results were highly congruent with the findings of Holt [14]. One of the key criteria for identifying *Streptomyces* is its spore morphology, which differs substantially between species. It has been discovered that the spores were arranged in chains, were rectiflexible, and had smooth spore surfaces, which were typical features of the genus *Streptomycetes* [15].

Molecular examination of the 16S rRNA gene of a selected isolate further supports this preliminary identification. According to the findings of a 16S rRNA phylogenetic analysis, the strain RVE129 is 99.65% related to *Streptomyces monomycini* NRRLB 24309. Similarly, various studies have noted the importance of 16S rRNA gene sequence matching as a technique for confirming the characterization of actinomycetes [5, 13, 16, 17, 23].

The characterization of major bioactive compounds has been published in prior studies using various approaches [23, 30–32]. The use of a range of techniques could aid in determining the nature of the bioactive antibiotic chemical isolation in the future. The purified antibiotic compound RVE-02 was soluble in ethanol, methanol, acetone, chloroform, petroleum ether, and DSMO but minimally soluble in water. Similar findings have previously been reported [23, 31]. The results showed that when the antibiotic compound was exposed to different temperatures and pH levels, it demonstrated relatively high temperature (45°C) and pH (pH = 9) stability. Thirumurugan and Vijayakumar [30] found that the *Streptomyces* sp. ECR77 polyketide antibiotic could be subjected to 45°C for 30 minutes with no loss of its antibacterial activity. The findings are also in agreement with those of Vijayakumar et al. [31], who observed that the antimicrobial compound of *Streptomyces* sp. VPTSA18 maintains its stability at pH 9 and 50°C. The result suggests that the antibiotic RVE-02, produced by *S. monomycini* RVE129, has potential applications in the pharmaceutical and food industry.

A bioactive metabolite fraction with an RF value of (RF = 0.73) was recovered via bioassay-guided fractionation and demonstrated significant bioactivity against Gram-positive and Gram-negative bacteria. Sanghvi et al. [23] have determined the antimicrobial potential of two bioactive fractions obtained from ethyl acetate extract of *Streptomyces werraensis* by thin-layer chromatography. The bioactive fraction-subjected UV spectroscopy has shown strong absorption maxima in the UV-visible spectrum at 277 nm and 327 nm, indicating the existence of conjugated systems in the chemical structure of the substance, such as unsaturated aldehydes, ester group, and aromatic ring compounds [32, 33] and the absence of polyenic structures [34]. These distinctive absorption bands at the wavelengths suggested that the purified compound could be a polyketide group of macrolide antibiotics. Similar observations were also made by other authors [20–23, 32–38].

The IR spectra data of purified fraction confirms the presence of carbonyls (ketone or ester group), hydroxyls, aromatic rings, and lactone moiety and the presence of prominent saturated carbons in various places, which are typical features of macrolide polyketide antimicrobial molecules [36]. Further, the IR spectra of purified fraction RVE-02 are almost identical to functional groups of the polyketide antimicrobial metabolite as reported for setamycin by Omura et al. [29], exhibiting peaks in the range of 3400–3500 cm^−1^, around 1660 cm^−1^, and 1717–1730 cm^−1^ which indicated hydroxyl, a hydrogen bonded carbonyl group and ketonic group or ester carbonyl, respectively. The chemical shift values of NMR ^1^H and ^13^C shown by the purified antibiotic RVE-02 were very similar to that of setamycin [39].

In general, the purified antibiotic compound produced by *S. monomycini* was identified using various analyses, and ^1^H and ^13^C NMR spectral data as well as a comparative evaluation of present data with Pubchem data was proposed that the antibiotic RVE-02 belongs to the polyketide macrolide antibiotic family to be setamycin with a molecular formula of C_44_H_65_NO_13_. The literature survey indicated that there were no reports on the production of polyketide antibiotics from *S. monomycini*. However, more research investigation is needed to elucidate the chemical structure of the antibiotic RVE-02.

The MIC values of the purified polyketide antibiotic RVE-02 against test bacteria ranged between 1.97 and 125 *μ*g/ml. The lowest MIC was recorded against *S. aureus* (1.97 *μ*g/ml). The findings of this study were comparable to those of broad-spectrum antibacterial bioactivity of polyketide antibiotics produced by *Streptomyces puniceus* strain AS13 [16], and *Streptomyces* sp. AP-123 [34].

The cytotoxicity result of the polyketide antibiotic RVE-02 demonstrated a strong antiproliferating effect on the HeLa cells with an IC_50_ value of 24.3 *μ*g/ml of the concentration. This is in line with the findings of Anuradha et al. [35], who reported that the cytotoxicity on HeLa cells had an IC_50_ value 26.20 *μ*g/ml as determined by the MTT assay. Earlier studies have also documented that the antimicrobial metabolite extracted from *Streptomyces* sp. causes antiproliferation activity and morphological changes in HeLa cells [16, 27, 28, 34]. These results showed that the antibiotic RVE-02 induced cell cytotoxicity against HeLa cell lines in a direct dose-response relationship where viability of cells was reduced, and a change in the shape of the cell was observed with increased antibiotic concentration in the treated cells.

## 5. Conclusion

This study demonstrated that the isolate RVE129 showed high similarity with *S. monomycini* based on phenotypic and genotypic characteristics. The antibiotic compound RVE-02 produced by strain can be identified and has close similarity with setamycin. This is the first report on the production of the polyketide antibiotic with antimicrobial and antitumour activity by the *S. monomycini* strain. The antibacterial bioactivity test indicated that the identified antibiotic RVE-02 is effective against a wide range of pathogens, including drug-resistant bacteria such as *S. aureus*. The antibiotic RVE-02 has antitumor activity against HeLa cells as tested *in vitro*. Thus, it is a promising candidate as an anticancer antibiotic. Current findings suggest the need for further investigations regarding the complete structure of the active compound and pharmacology of RVE-02.

## Figures and Tables

**Table 1:** The growth characteristics of the *Streptomyces* sp RVE129.

Culture medium	Growth pattern	Aerial mycelium	Substrate mycelium	Diffusible pigments
Tryptone yeast extract agar (ISP-1)	++	Grey	Pale yellow	—
Yeast extract malt extract agar (ISP-2)	+++	White	Brown	—
Oat meal agar (ISP-3)	++	White	Grey	Brown
Inorganic salt starch agar (ISP-4)	+++	Brown	Light ivory	—
Glycerol asparagines agar (ISP-5)	++	Grey	Greyish white	—
Peptone yeast extract iron agar (ISP-6)	+++	Grey	Dark grey	—
Tyrosine agar (ISP-7)	+	Cream	Brown	—
Starch casein agar	+++	White	Whitish brown	—

+++: good growth; ++: moderate growth; +: poor growth; −: no diffusible pigment.

**Table 2:** Morphological, biochemical, and physiological characteristics of strain RVE129.

Characteristics	Result	Characteristics	Result
*Microscopic examination*		Indole	—
Gram reaction	+	Voges-Proskauer	—
Spore chain morphology	Rectiflexible	*Carbon utilization*	
Motility	Nonmotile	Inositol	—
Growth	Aerobic	Mannose	+
*Physiological characteristics*		Xylose	±
Temperature range for growth	20-40°C	Adonitol	±
Optimum temperature	30°C	Fructose	+
pH range for growth	4 to 10	Sucrose	+
Optimum pH for growth	7	Sorbitol	+
NaCl tolerance range	0-5%	Trehalose	+
Optimum NaCl (%) for growth	2.5%	Arabinose	—
*Biochemical characters*		Lactose	+
Melanin production	—	Galactose	+
Nitrate reduction	—	Maltose	+
Catalase production	+	Mannitol	+
Urease production	+	Glucose	+
Esculin degradation	+	Cellulose	±
H_2_S production	+	Starch	+
Citrate utilization	+	Fructose	**+**
Casein hydrolysis	+	Rhamnose	**—**
Oxidase production	—		
Gelatin hydrolysis	+		

+: positive; ±: doubtful/poor; −: negative.

**Table 3:** NMR spectra data of antibiotic compound RVE-02.

Position	*δ*C of purified compound	*δ*C setamycin [29]	Position	*δ*C of purified compound	*δ*C setamycin [29]
1	167.48	167.3	21	76.28	76.8
2	141.17	141. 2	22	40.0	40.0
2-OCH	60.02	59. 9	23	75.51	75.5
3	133.41	133.4	24	28	27.9
4	133.39	133.3	25	12.35	12.3
5	143.02	143.0	26	7.23	7.1
6	36.64	36.7	27	13.9	14.0
7	81.12	81.2	28	17.42	17.3
8	37.17	37.1	29	10.00	9.8
9	41.22	41.2	30	20.2	20.2
10	142.80	142.8	31	21.58	21.6
11	133.22	133.0	32	14.32	14.3
12	125.35	125.2	33	20.92	21.0
13	127.22	127.0	C-1′	164.29	164.3
14	70.16	70.24	C-2′	133.59	133.6
14-OCH	55.54	55.5	C-3′	133.04	133.0
15	77.36	77.2	C-4′	163.6	163.6
16	38.20	38.2	C-5′	115.02	114.9
17	82.18	82.2	C-6′	175.24	175.2
18	42.05	42.4	C-7′	26.00	25.8
19	98.42	98.2	C-8′	32.17	32.2
19-OH	—	—	C-9′	198.00	197.7
20	40.08	40.0	9′ OH	—	—

**Table 4:** Bioactivity potential of antibiotic RVE-02 against different pathogenic bacteria.

Test organisms	MIC (*μ*g/ml)	Erythromycin (*μ*g/ml)
*Staphylococcus aureus* ATCC-259233	1.97	1.97
*Staphylococcus epidermidis* ATCC-12228	3.95	1.97
*Salmonella typhi* ATCC-13311	31.25	7.90
*Pseudomonas aeruginosa ATCC-27853*	125	31.25
*Kelbsiella pneumonia ATCC-700603*	125	62.5
*Escherichia coli* ATCC-25922	62.5	7.90
*Salmonella typhi* ATCC-14028	62.5	7.90

## Data Availability

Further data related to this study can be made available upon request from the corresponding author.
